# Prospects of nanoparticle-based radioenhancement for radiotherapy

**DOI:** 10.1039/d3mh00265a

**Published:** 2023-08-03

**Authors:** Lukas R. H. Gerken, Maren E. Gerdes, Martin Pruschy, Inge K. Herrmann

**Affiliations:** a Nanoparticle Systems Engineering Laboratory, Institute of Energy and Process Engineering (IEPE), Department of Mechanical and Process Engineering (D-MAVT), ETH Zurich Sonneggstrasse 3 8092 Zurich Switzerland ingeh@ethz.ch +41 (0)58 765 7153; b Particles-Biology Interactions Laboratory, Department of Materials Meet Life, Swiss Federal Laboratories for Materials Science and Technology (Empa) Lerchenfeldstrasse 5 9014 St. Gallen Switzerland; c Karolinska Institutet Solnavägen 1 171 77 Stockholm Sweden; d Laboratory for Applied Radiobiology, Department of Radiation Oncology, University Hospital Zurich, University of Zurich Winterthurerstrasse 190 8057 Zurich Switzerland

## Abstract

Radiotherapy is a key pillar of solid cancer treatment. Despite a high level of conformal dose deposition, radiotherapy is limited due to co-irradiation of organs at risk and subsequent normal tissue toxicities. Nanotechnology offers an attractive opportunity for increasing the efficacy and safety of cancer radiotherapy. Leveraging the freedom of design and the growing synthetic capabilities of the nanomaterial-community, a variety of engineered nanomaterials have been designed and investigated as radiosensitizers or radioenhancers. While research so far has been primarily focused on gold nanoparticles and other high atomic number materials to increase the absorption cross section of tumor tissue, recent studies are challenging the traditional concept of high-*Z* nanoparticle radioenhancers and highlight the importance of catalytic activity. This review provides a concise overview on the knowledge of nanoparticle radioenhancement mechanisms and their quantification. It critically discusses potential radioenhancer candidate materials and general design criteria for different radiation therapy modalities, and concludes with research priorities in order to advance the development of nanomaterials, to enhance the efficacy of radiotherapy and to increase at the same time the therapeutic window.

## Abstract 2

Inorganic nanomaterials show promising potential for radiotherapy enhancement. This article provides a concise summary of mechanisms involved, materials designs and future prospects of nanoparticle-based radioenhancement for precision medicine.

## Graphical abstract

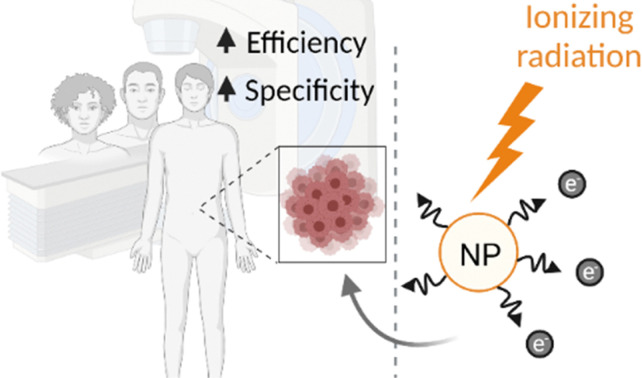

## 1. Introduction

**Fig. 1:**
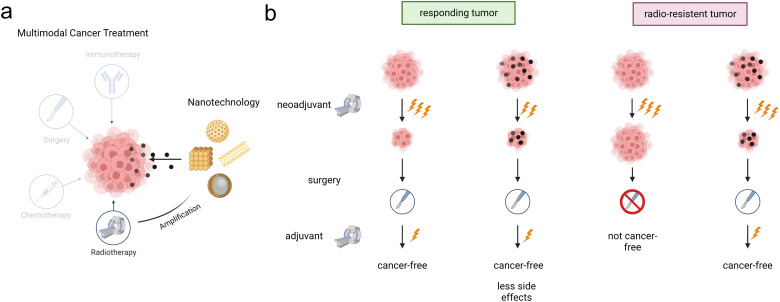
Schematic illustration of the potential integration of nanotechnology into cancer treatment (a), and a representation of beneficial treatment settings using radioenhancer nanoparticles (b). Figure created with BioRender.com.

Approximately 50% of all cancer patients have an indication for radiotherapy at least once during the course of their disease,^1–3^ with an absolute number of patients steadily increasing assuming overall cancer rates remain unchanged.^4^ Radiotherapy can be administered to cure cancer or relieve cancer symptoms, either as monotherapy or in multimodal cancer treatment in combination with surgery, chemotherapy or immunotherapy (Fig. 1).^5^ It is either employed in a neoadjuvant setting to shrink the tumor before surgery, or in an adjuvant setting to destroy left-over cancer cells or both. Radiotherapy effects can be further enhanced by the addition of (nanomaterial-based) radioenhancers, extending the therapeutic window by increasing efficacy and reducing side effects.^6^ Advanced radiation treatments and radiotherapy techniques (*e.g.* particle- *vs.* photon-radiotherapy; intensity-modulated radiotherapy and volumetric arc therapy) are nowadays established in clinical practice and are well suited for planning and delivering of radiotherapy with conformity.^7,8^ Despite these technical advances in modern, image-guided, adaptive radiotherapy, radiation toxicity to co-irradiated adjacent normal tissue still determines and limits the maximal dose that can be applied to a tumor, and is a most critical limitation of contemporary radiotherapy.^3^ To reduce healthy tissue damage in organs at risk and to overcome radioresistance of tumors (*e.g.* due to a hypoxic tumor environment),^9–12^ new strategies rendering the tumor tissue more susceptible are sought after.

Radiosensitizers and radioenhancers in the form of high-*Z* metal nanoparticles (NPs) deposited in the tumorous tissue have recently attracted considerable attention as an alternate therapeutic route that can overcome these limitations and widen the therapeutic window. A pioneering study in 2004 demonstrated control of a malignant tumor *in vivo* by the administration of gold nanoparticles prior to radiotherapy.^13^ Presently, the radioenhancing effect of NPs is widely accepted, with two candidate materials, AGuIX® (NH TherAguix, Lyon, France) and CE-certified NBTXR3/Hensify® (Nanobiotix, Paris, France), in clinical evaluation.^14–16^ In addition to widely studied metal and metal oxide radioenhancer nanoparticles, new material design strategies offer the possibility of (multimodal) combination treatments, paving the way to even more effective cancer treatments in the future. For example, drug-loadable metal–organic frameworks (MOFs) can generate toxic reactive oxygen species (ROS) during irradiation, offering new prospects for combination treatments.^17,18^ Although a vast number of different nanomaterials have been explored as radioenhancers, the translation to clinical application is slow.^19^ Current major translational barriers include the scalability of high-quality nanoparticle manufacturing and the significant knowledge gaps in nanoparticle design, effectiveness and biological activity.^19,20^ In particular, the limited understanding of nanoparticle-based radioenhancement precludes rational material designs and limits the full exploitation of the available materials design space. The scarcity of comparative studies and benchmarking prevents relative comparison of candidate materials and subsequent rational selection of the best performing candidate.^21–23^ The deficient understanding of radiotherapy enhancement mechanisms and nanomaterials toxicity additionally inhibits the design of optimal candidate materials.^19^ This gap in fundamental mechanistic understanding implies that potentially considerable gains in performance can be achieved through rationally designed materials and appropriate material selection based on irradiation conditions (external *vs.* internal radiotherapy, photons *vs.* protons, beam energy).

In the following, we provide a concise summary of the current knowledge on radioenhancement mechanisms. Key aspects of physical, chemical and biological mechanisms and their quantifications during radiation therapy with and without nanoparticles are discussed. Nanoparticle radioenhancer material candidates and their radioenhancing properties from a preclinical perspective and their clinical progress are presented. From this, we deduce key materials design criteria and corresponding considerations for future research in this emerging field.

## 2. Principles of radiotherapy: physical, chemical and biological responses to ionizing radiation

To delineate the complex processes in between initial energy deposition and biological responses to irradiation, four stages can be defined: (i) the physical, (ii) the physico-chemical, (iii) the chemical, and (iv) the biological stage.^9^ At the physical stage, ionizing radiation travels in the form of particles or electromagnetic waves through the target medium and excites or ionizes the molecules in its path. Initial physical processes in local track regions happen within the first ∼10^−15^ s.^10^ Dissociative decay, auto-ionization, thermalization, or solvation characterize the physico-chemical stage (time scale ∼10^−15^–10^−12^ s). The following chemical stage (∼10^−12^–10^−6^ s) is associated with the diffusion and production of initial or new chemical species, resulting in a homogenous distribution of radiolysis products. Since the water content in tumors, tissues, and organs typically is around 70–85%,^11^ water radiolysis takes on an important role in the cellular response especially to ionizing radiation at the low LET (linear energy transfer) level of clinically relevant photon and proton irradiation. The physical and chemical processes related to water radiolysis are very well studied: irradiated water after the initial physical stage contains excited H_2_O* and the ionized species H_2_O^+^ and e^−^. During the physico-chemical stage, H_2_O* molecules can dissociate to produce ˙H and ˙OH, H_2_ and ˙O, and H_2_O^+^ and e^−^ species or radicals. Further processes such as ion–molecule reactions (H_2_O^+^ + H_2_O) and/or electron and ion hydration lead to the production of (H_3_O^+^)_aq_, (e^−^)_aq_, H_3_O^+^, and ˙OH species. During the chemical stage, the initially formed radiolysis products diffuse and interact with each other to create OH^−^, H_2_O_2_, and additional H_2_ while consuming some of the initially generated ˙OH, ˙H, H_3_O^+^, and (e^−^)_aq_ species.^9,12^ Yields of the different water radiolysis products can be found in several simulation reports and are dependent on the ionizing source used.^12–14^

At the biological stage, the responses can be determined at different spatial and temporal resolution with some biological processes manifested only months, years or decades after irradiation (*e.g.* chronic normal tissue toxicities and secondary malignancies), phenomena which are beyond the focus of this review. The immediate biological damage can be induced by direct or indirect irradiation effects. Direct damage, caused by incident photons, charged particles and their subsequently generated electron splashes, is only detrimental when vital elements of the cell are directly affected. Critically important cellular components for ionizing radiation-induced damage include the DNA, the cellular membrane, and essential cell organelles (mitochondria,^15^ endoplasmic reticulum, ribosomes, and lysosomes).^16^ Low LET photon and proton irradiation only induce a small proportion of (water radiolysis-independent) direct biological damage, while its relative importance increases with increasing LET radiation.^17^ The dominant indirect effect of low LET irradiation is mainly induced through water radiolysis products, which can cause DNA breaks and complex chromosomal aberrations, protein denaturation, cell membrane disruption, enzyme inactivation, and DNA or RNA mutations.^9^ An important and dominating role driving indirect damage is attributed to ˙OH radicals, which are considered the most powerful oxidant among the water derivatives.^8^ In fact, it has been shown through the use of hydroxyl scavengers that 60–90% of cellular radiation damage can be attributed to ˙OH radicals.^17–20^ This hydroxyl-mediated indirect damage action is strongly dependent on the oxygen partial pressure (*p*O_2_) in the tumor microenvironment.^19^ A commonly used explanation for the observed oxygen enhancement effect is that free-radical-induced DNA damage is “fixed” in the presence of molecular oxygen (“oxygen fixation theory”), thereby making it permanent.^16^ Additionally, highly deleterious oxidizing radicals (superoxide, ˙O_2_^−^, and hydroperoxyl radicals, ˙OOH) are created during the chemical stage of radiolysis in the presence of molecular oxygen.^14^ Thus, the presence of oxygen in the target medium has a crucial impact on the success of the radiotherapy and tumor hypoxia represents a major radioresistance mechanism. Overall, the complex radiation response on the cellular and (patho-) physiological level have been described as the 6 *R*s of radiobiology, where a change in any one of the *R*s can increase or decrease the net therapeutic effect of fractionated radiotherapy (see below).^22,23^

## 3. Nanoparticle radioenhancement mechanisms

**Fig. 2:**
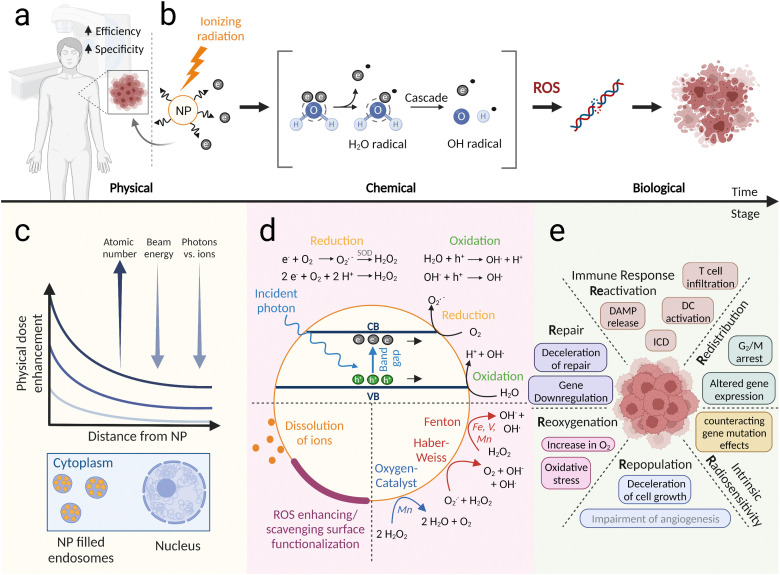
Mechanisms of nanoparticle radioenhancement. (a) Metal or metal oxide nanoparticles may be inserted into tumorous tissue, *e.g. via* injection or intravenously *via* the EPR effect, and act to enhance the efficiency and specificity of the applied radiation (*e.g.* X-rays, protons). (b) The mechanism of action of nanoparticle radioenhancers can be divided into three stages: a physical, a chemical and a biological stage. In the physical stage, ionizing radiation leads to the ejection of secondary electrons which can then, in the chemical stage, interact with other molecules, *e.g.* water, causing the creation of ROS. In the biological stage, the created ROS interacts with the components of the cell (*e.g.* DNA), eventually triggering cell death. (c) and (d) Detailed view of the three stages: (c) physical dose enhancement by NPs located in endosomes decreases with increasing distance from the NP, as well as beam energy and when photons instead of ions are used as the modality. The higher the atomic number of the particles, the stronger the physical dose enhancement. (d) Chemically, NPs can contribute to the creation of ROS through photocatalytic activity (top), surface effects (bottom left) or the action of oxygen catalysts (*e.g.* Mn) or Fenton and Haber–Weiss reactions (*e.g.* Fe) (bottom right). (e) Biological nanoparticle enhancement mechanism can be viewed in context of the 6 *R*s of radiobiology. Figure created with BioRender.com.

Nanoparticles that can increase the effect of radiotherapy by altering one or more of the aforementioned response stages can be classified as radioenhancers or radiosensitizers (Fig. 2a). While “radiosensitizer” defines any substance that sensitizes cells to radiation therapy, and has thus a more general meaning, the term “radioenhancer” implies an amplification role, such as delivering a higher dose to the tumor^24^ or increasing the chemical ROS species. Therefore, in this review we use the term radioenhancer for nanoparticles that are able to increase ionizing dose or ROS inside the tumor, while radiosensitizers are nanoparticles that biologically sensitize cells to ionizing radiation.

The processes underlying nanoparticle radioenhancement can be divided into different stages (Fig. 2b): excitation and ionization of the NPs (physical stage), generation of reactive oxygen species (ROS) in the surrounding medium (chemical stage), and biological implications of increased damage on a cytoplasmic or nuclear level (biological stage). Furthermore, NPs can modulate biological processes, thereby sensitizing cells to ionizing radiation and increasing the effects of radiotherapy. The exact mechanisms through which NPs enhance ionizing radiation stage-dependent processes on the physical, chemical, and biological level are influenced by the nanoparticle composition and are the subject of ongoing research.

### 3.1 Physical enhancement

The physical interaction between NPs and ionizing radiation depends significantly on the modality of radiation used and its energy (Fig. 2c). Low energy kiloelectronvolt photons (<500 keV) interact with NPs *via* photoelectric absorption, resulting in the ejection of a photoelectron from the K, L, or M shell and the subsequent emission of fluorescence and Auger electrons.^25^ Regarding secondary electron emission after ionization of a NP, Monte Carlo simulations have shown that the NP dose enhancement decreases exponentially with increasing distance from the NP surface.^26^ Auger electrons contribute to enormous low-range (∼10 nm) dose enhancement, while electrons of higher energy (photo- or Compton electrons) contribute to micrometer-range (up to 30–40 μm) enhancement, which can lead to direct DNA damage.^26,27^ The photoelectric effect probability scales roughly with *Z*^4^/*E*^3^, rendering high-*Z* materials attractive candidates for radioenhancement using low-energy photon beams.^28^ However, clinically relevant applications typically use higher energies, and megaelectronvolt (MeV) photon interactions with NPs predominantly result in Compton scattering or pair production with interaction probabilities only linearly proportional to *Z*.^25^ Physical dose enhancement for MV photons was simulated to be negligible in cellular scenarios even for high-*Z* NPs,^29^ although very localized nanoscopic dose enhancement around gold nanoparticles remains a topic of discussion.^30–32^ For a highly localized dose to grant a therapeutic advantage from secondary species emission, NPs can be functionalized in order to target sensitive subcellular localizations. It has been shown that targeting the cells’ nucleus^33,34^ or mitochondria^35–37^ can indeed lead to enhanced X-ray treatment efficiency.

The major difference between photon- and particular proton-based radiotherapy at the macrolevel is their differential spatial distribution of energy deposition. Photon beams have the highest dose deposition close to the entrance surface and continuously deposit dose along the entire path throughout the tissue. Generally, this involves healthy tissue being co-irradiated proximally as well as distally of the target volume. In contrast, proton beams commonly deposit a lower dose in the entry field, and maximum dose deposition occurs within the so-called Bragg peak at a depth defined by the velocity of the applied protons. Behind this Bragg peak region – or Spread-Out Bragg Peak (SOBP) in clinical applications – no significant dose is deposited. The reduced volume of healthy tissue exposed to intermediate and low doses of proton radiotherapy results in reduced co-irradiation of dose-limiting organs at risk (OAR) such as brain stem, spinal cord, oral cavity, or the optic nerve and subsequently also a reduced risk of secondary malignancies in these co-irradiated organs. A physical characteristics of proton radiotherapy is the increase in LET towards the distal end of the spread-out Bragg Peak (SOBP).^38,39^ Irradiation with elevated LET induces more complex respectively clustered DNA lesions than yielded by conventional low LET photon irradiation. Utilizing proton-based instead of photon-based external beam irradiation alters the beam's interactions with NPs.^40^ These positively charged subatomic particles deposit energy through ionization and excitation of matter *via* Coulombic interactions.^41^ While the beneficial interaction of protons with NP radioenhancers has been demonstrated,^40,42–45^ a mechanistic understanding at the nanoscale level is very much under debate. Kim *et al.* first attributed the observed dose enhancement effects to particle-induced X-ray emission (PIXE).^42^ This explanation, along with enhancement by particle-induced gamma-ray emission (PIGE), was later rejected. Other mechanisms were considered as being more relevant, such as a very local (<100 nm range) secondary electron emission through Coulombic interactions of the protons with the NP.^43,46^ Indeed, Monte Carlo simulations have indicated a potential proton dose enhancement from small nanoparticles within <10 nm distance stemming from excess Auger electrons.^47^ A simulation with 1.3 MeV protons showed, that the yield of such secondary electrons from titanium surfaces could even be higher than from high-*Z* nanoparticles.^48^ Auger electrons have the ability to form ROS by radiolysis of water molecules.^49^ Other reports, using a model that underestimates the low-energy electron emission spectra, have concluded that physical dose enhancement from proton irradiated nanoparticles is negligible, especially when compared to kV photons.^29,50^ The accurate reproduction of the low-energy electron emission spectra remains a key challenge in simulations.^51,52^ It remains to be elucidated, if the nanoscale physical effects contribute to enhanced ROS creation observed during nanoparticle irradiation with protons, or whether they are solely created by catalytic effects.^40,46,53–55^

#### Quantifying physical dose enhancement

To assess and gain insights into physical dose enhancement by various NPs, macroscopic calculations^56^ and Monte Carlo simulations^27,29,30,57^ can be employed to predict the physical NP dose enhancement effect at the nanoscopic to macroscopic scale. These can be complemented by experimental observations of physical dose enhancements in acellular (cell-free) systems, although such observations are generally more complicated to perform and interpret. A few studies are available and generally in line with simulations. For instance, nanoparticle-impregnated dl-Alanin wax pellets have demonstrated dose enhancements of approximately 60% (for kV X-rays), 10% (for 10 MV X-rays), and ≤5% (for proton and electron beams) for gold nanoparticles.^58^ Nanoparticle-loaded water-equivalent PRESAGE dosimeters have been used to show physical dose enhancement of Au, Bi_2_S_3_, and Bi NPs, with a higher dose enhancement from kV X-rays (12–32%) than from a clinical 6 MV X-ray beam (2–5%).^59^ The use of PRESAGE dosimeters is limited, however, due to the optical readout method, which is susceptible to NP interference, especially at higher NP concentrations. Alternative methods using MRI or CT as readout methods are found in other polymer dosimeters,^60^ such as MAGIC (Methacrylic and Ascorbic acid in Gelatin Initiated by Copper),^61,62^ MAGAT (methacrylic acid gelatine and tetrakis (hydroxymethyl) phosphonium chloride),^63^ or nPAG (normoxic polyacrylamide).^64^ Most of the studies using gel dosimeters, or similar methods, focus on dose enhancement with Au NPs. Thus, a systematic comparison of different NPs in such systems is missing. A more detailed knowledge of the system, coupled with Monte Carlo simulations, could provide useful understanding of the physical mechanisms of nanoparticle dose enhancement.

### 3.2 Chemical enhancement

Generally, *in vitro* and *in vivo* dose enhancement findings have greatly exceeded predictions made by simulations of physical enhancement under clinically relevant photon or particle beam irradiation conditions, suggesting that chemical and biological effects play crucial roles.^51,65–67^ Increased ROS generation has been suggested as a major driving force of NP-enhanced radiation damage.^68^ Several *in vitro* studies have shown that ROS quenchers decrease the nanoparticle-induced enhancement effect.^55,69,70^ The mechanisms of NP-based ROS formation in cells, however, are complex and occur on different levels. NPs can enhance ROS formation through secondary electron emission, which is the chemical follow-up stage to the aforementioned physical dose enhancement processes. Furthermore, ROS formation can be increased by catalytic nanoparticle surface processes, such as lowering the ionizing potential of surrounding molecules^71^ or acting as an electron or hole donor (Fig. 2d).^72,73^ The latter process is commonly found in semiconductor nanoparticles and is most effective when the potentials of the valence and charge bands are suitable for water splitting.^74,75^ In a comparison of different semiconductor nanomaterials, our group has shown that TiO_2_ and WO_3_ nanoparticles have higher ROS generation ability compared to HfO_2_, TiN and SiO_2_ during the irradiation with X-rays and protons.^55^ The nanoparticles also generated more ROS under X-ray compared to proton irradiation. Other chemical enhancement mechanisms include upregulation of the local oxygen concentration *via* exogenous oxygen delivery or catalytic decomposition of H_2_O_2_ (to overcome hypoxia) or turning H_2_O_2_ into more toxic ˙OH radicals *via* Fenton or Haber–Weiss reactions (Fig. 2d).^68,76,77^ Successful modulation of the hypoxic tumor microenvironment has been demonstrated using a variety of particles (such as perfluorocarbon-, hemoglobin-, metal–organic framework-, Mn-, Pt-, or Fe-based nanoparticles), and a comparison of their oxygenation efficiency can be found in a review by Li *et al.* (2021).^78^ Fenton agents for cancer therapy include Fe-based nanomaterials or redox-active transition metals (such as Cu, Mn, Ag, V, Co, and W),^76^ and the reader is referred to reviews from Cao *et al.* (2021) and Zhang *et al.* (2021) for a perspective on Fenton/Fenton-like agents or ROS elevating nanomedicines.^77,79^ Interestingly, nanoparticle surface bound ions as well as released ions can contribute to Fenton reactions. For example, the controlled release of Fe ions from FePt NPs in lysosomal conditions (acidic pH), led to more increased ROS formation by the catalytic decomposition of H_2_O_2_.^80^ In a study with iron oxide NPs and iron ions, ROS concentrations were increased by ions and by the NP surface after X-ray exposure.^81^ In another study with CuO NPs and dissolved Cu ions, it was found that *via* Fenton-like and Haber–Weiss reactions the NP surface contributed to a significant portion of the observed amount of ROS and plasmid DNA damage.^82^ Such reactions with copper ions have also been shown to enhance ROS and cell death after X-ray exposure.^83,84^ The functionalization of NP surfaces can provide an additional strategy to modulate ROS generation (Fig. 2d). However, since surface modifications can decrease the surface reactivity inhibiting ROS generation,^85^ they have to be chosen carefully. For example, it was shown for different PEG and human serum albumin functionalizations of Au NPs, that the amount of ˙OH radical production and plasmid DNA damage during X-ray irradiation dramatically decreased with the number of atoms in the coating.^86^ On the contrary, surface functionalization can be used to enhance charge separation and transfer and improve catalytic reactions,^87^ or allow the creation of other highly reactive species such as singlet oxygen (^1^O_2_) which can be produced by *e.g.* porphyrins.^88^ Ideally, a nanoparticle coating has several beneficial therapeutic effects. For instance, surface coatings of mixed-phase iron oxide NPs resulted in more reactive surfaces during X-ray exposure while also improving their biocompatibility.^89^

#### Quantifying chemical enhancement

Several methods are available to detect ROS such as electron spin resonance (ESR/EPR), fluorescent/chemiluminescent probes or proteins, chromatography/spectrophotometry methods, and electrochemical biosensors, each having its advantages and disadvantages.^90^ Fluorophores or chemiluminescent probes provide an easy means of measuring irradiation-induced ROS in cells or in acellular nanoparticle solutions using a microplate reader or fluorescence microscope. To measure ROS in acellular nanoparticle solutions, assays need to be optimized and nanoparticle–fluorophore interferences need to be understood and minimized.^91–93^ Frequently used ROS fluorophores include 2,7-dichlorofluorescein diacetate (DCFDA; unspecific ROS or nitrogen species),^55,91,94^ coumarin-3-carboxylic acid (3-CCA)/7-hydroxycoumarin (˙OH specificity),^95–97^ Amplex® Red (H_2_O_2_),^98^ dihydrorhodamine (DHR; ˙O_2_^−^, ONOO^−^, ˙OH specificity),^99,100^ 3-(*p*-aminophenyl) fluorescein (APF; ˙OH, ONOO^−^, OCl^−^ specificity),^101,102^ dihydroethidium (DHE; ˙OH, ˙O_2_^−^ specificity),^96,102^ singlet oxygen sensor green (SOSG; ^1^O_2_ specificity),^96^ CellROX (unspecific ROS),^103^ and MitoSOX (mitochondrial ˙O_2_^−^ specificity).^68,104,105^ Indirect harvesting methods can also show the importance of certain radicals during nanoparticle-enhanced radiation therapy. Dimethyl sulfoxide (DMSO), for instance, has been applied as a hydroxyl radical scavenger during the irradiation of cells with and without nanoparticles to show the importance of those radicals in successful radiotherapy and nanoparticle enhancement mechanisms.^55,69,70,106^

### 3.3 Biological sensitization mechanisms

In radiotherapy, tumor control and normal tissue toxicity are determined by the total dose, fraction size, number of fractions, time between fractions and the overall treatment time. Increasing the fraction size and decreasing the time between fractions and the overall treatment time improve tumor control, but at the same time, decrease the normal tissue protection effect of fractionation. Mechanistically, the differential tumor and normal tissue responses could be linked to the so-called 5 *R*s of radiotherapy, namely repair, redistribution, repopulation, reoxygenation and intrinsic radiosensitivity. These “hallmarks of radiotherapy” group the plethora of molecular processes induced by fractionated radiotherapy into biologically relevant concepts. Lately and based on the advancement of immunotherapy and single high dose radiotherapy, an additional 6th *R* has been proposed, which is the reactivation of anti-tumor immune response. These *R*s of radiotherapy have often been used as guidelines for the development of novel combined treatment modalities, to understand differential response patterns to low dose fractionated *versus* hypofractionated radiotherapy and to different radiation modalities. Nanoparticle radioenhancers will sensitize both tumor and normal cells to ionizing radiation and it will be important to identify which *R* of radiotherapy and related biological process is most affected or could be exploited to further increase a therapeutic window and to even design a personalized regimen with NPs (see Fig. 2e).

#### Repair

Human cells have developed sophisticated DNA damage repair machineries to guarantee genomic integrity after an insult. NPs have been shown to interfere with a cell's DNA repair system, reducing the capability of cancer cells to respond to ionizing radiation-induced DNA damage and thereby increasing treatment efficacy. Au NPs may be capable of slowing down the repair machinery^107–109^ (as opposed to inducing more DNA double-strand breaks) and downregulating DNA repair genes such as BRCA1,^110^ MSH3,^111^ and MRE11A^111^ [see also review by Penninckx *et al.* (2020)^112^ for details]. How exactly Au NPs exert this influence is unclear and requires further investigation. Effects on DNA repair have also been observed with other materials. For instance, Wojewodzka *et al.* (2011) reported that Ag NPs delayed repair of DNA damage inflicted by kV X-rays in human HepG2 cells, while TiO_2_ NPs were less effective.^113^ However, a prolonged, 2 month exposure to TiO_2_ has been shown to impair DNA repair processes in A549 cells, suggesting that chronic exposure to TiO_2_ sensitizes cells towards genotoxic agents.^114,115^ In another study, Ti nanotubes were shown to decrease DNA repair efficacy in human glioblastoma cells.^116^ A study by Štefančíková *et al.* (2016) indicated that Gd-based NPs (AguIX), currently in phase 2 clinical trials, had no impact on the creation of DNA lesions or DNA repair in glioblastoma cells under irradiation with gamma rays.^117^ Nanomaterials can be categorized into 4 main groups in an attempt to forecast the modes of action not only towards apical toxicity effects, but also towards impacting DNA repair processes: (i) soluble nanomaterials that would release metal ions in their surrounding environment (*e.g.* Ag, ZnO, CuO, or CdSe NPs); (ii) biopersistent high aspect ratio nanomaterials which show fibre-like effects (*e.g.* carbon nanotubes); (iii) passive nanomaterials that carry a non-reactive surface; and (iv) active nanomaterials with reactive surface properties that may activate or inversely inactivate biological molecules and reactions (*e.g.* TiO_2_ or CeO_2_).^118,119^ One common mechanism influencing DNA repair, that can apply to nanomaterials of all 4 groups, is the sequestration of DNA repair proteins in the nanomaterial protein corona.^118^

#### Redistribution

A classic rationale for the use of fractionated radiotherapy is the differential radiosensitivity of cancer cells in different phases of the cell cycle, which may be exploited to synchronize cancer cells into radiosensitive phases.^120^ Interestingly, nanoparticles can also lead to cell cycle synchronization. However, this might be highly cell specific, and might also be influenced by nanoparticle size or surface. For instance, some groups could not show that Au NPs have significant cell cycle effects,^109,121–123^ while others could demonstrate an NP-induced shift of the cellular cell cycle distribution into a more radiosensitive cell cycle phase thereby increasing the response to irradiation.^124–126^ Altered gene expression, for instance of cyclins and checkpoint inhibitors, might be one of the underlying mechanisms,^124,125,127^ but a detailed understanding is currently lacking. In addition to Au NPs, Gd NPs^128^ and Ag NPs^129^ were also reported to display G2/M phase arrest in F98 rat glioma and U251 human glioblastoma cells, respectively.

Last but not least, a NP-dependent increase of ROS-generation in response to irradiation might also lead to enhanced levels of DNA damage *per se*, with subsequently more cytotoxic chromosomal aberrations and differential cell cycle checkpoint activation. Corrupted DNA repair machineries and cell cycle checkpoints, which are often abundant in tumor cells but not in untransformed cells, might thereby contribute to an increased therapeutic window, and could be further enhanced on a personalized level. For example, DNA damage in response to high-energy proton irradiation requires different DNA repair machineries than in response to photon irradiation,^130,131^ which could be exacerbated in combination with NPs.

#### Repopulation

Tumor control in response to fractionated radiotherapy might not be reached in highly proliferating tumors due to (accelerated) repopulation, an event in which tumor cells (possibly tumor stem cells) rapidly proliferate after receiving sublethal irradiation.^132^ NPs may be employed to decelerate cell growth and to hamper repopulation. Au NPs have been shown to negatively affect proliferation of certain cell lines, for instance by cell cycle arrest, and revascularization, which could be mechanistically linked to direct binding to heparin-binding growth factors thereby impeding VEGF-dependent angiogenesis,^133–135^ and limiting the secretion of inflammatory cytokines (IL-6, IL-1β).^136^ In contrast, repopulation could also lead to the diffusion of administered NPs in the tumor, as shown for Au NPs,^137,138^ which in turn can limit NP-mediated radiosensitization. Furthermore, it will be important to investigate to which extent NPs will interfere with the proliferation of normal tissue to manage acute normal tissue toxicities.

#### Reoxygenation

As discussed above and independent of the cellular genotype, hypoxic cells are up to three-fold more radioresistant than normoxic cells, which is explained by the oxygenation fixation theory. This theory implies that radiation-induced free radical sites in the DNA are chemically derivatized (“fixed”) in the presence of oxygen so that they cannot be repaired and accumulate, leading to an enhanced rate of cell death. Furthermore, normoxic conditions favor the generation of reactive oxygen species, in particular superoxide and hydroperoxyl radicals, in response to ionizing radiation which eventually results in a higher amount of DNA damage.^14,139^ Fractionated irradiation exploits the phenomenon of (iterative) reoxygenation in which hypoxic cells become reoxygenated and subsequently more radiosensitive, *i.e.* a dose of ionizing radiation will preferentially kill the normoxic cell population and the remaining cell population with a higher relative proportion of hypoxic cells will become reoxygenated (and more radiosensitive) by the microenvironment thereafter to be killed by the next dose of a fractionated treatment schedule. The presence of oxygen and the generation of ROS are also important for NP radioenhancement. For instance, results from Au NP radioenhancement studies have shown that enhancement is greater under normoxia than hypoxia,^108,140^ and dampened in the presence of radical scavengers.^69,141^ Though Au NPs alone cannot reoxygenate tissue, they can be coupled to an oxygen reservoir such as liquid perfluorooctyl bromide (PFOB), which rapidly releases O_2_ upon ultrasound (US) treatment, in order to relieve hypoxia, generate more ROS and prevent DNA repair, leading to a higher radiotherapeutic effect.^142^ In contrast, other inorganic nanomaterials can generate O_2_ by dissolution (*e.g.* CaO_2_)^143^ or *via* an enzyme-like decomposition of H_2_O_2_. In the latter case, MnFe_2_O_4_ nanoparticles have successfully been used to increase the local *p*O_2_*via* the decomposition of H_2_O_2_ in *in vivo* tumor tissues to render radiotherapy more efficient.^144^ Similar, catalase-like, effects were observed with a variety of materials, *e.g.* CeO_2_^145^ and V_2_O_5_^146^ (for details see review by Ruan *et al.* (2021)^147^). Targeting hypoxic cancer cells and inducing reoxygenation are important steps in overcoming radioresistance, and more emphasis should be placed on developing appropriate materials and unraveling their underlying mechanisms.

#### Intrinsic radiosensitivity

On the preclinical level, it is very well established that the genetic make-up of tumor cells and normal tissue influence the treatment response to irradiation. However, insights on the genetic background and its impact to a differential radiation response – have not yet developed into clinical radiotherapy strategies on the personalized level.^148^ Irradiation may induce different modes of cell death in different tumor entities in dependence of the genetic and normal tissue background they derive of. A widely accepted hypothesis suggests that the presence of acquired mutations can render cells more or less prone to programmed cell death, with a prominent example being p53.^149^ Therefore, nanoparticles that are able to modify the cell intrinsic radiosensitivity through inhibition of specific targets responsible for treatment resistance of the cancer cell would be interesting for therapy. Au NPs have been implicated in inducing apoptosis, for instance by activation of caspases and, as a result, triggering the rupture of the mitochondrial membrane and the release of cytochrome *c*,^150^ thereby aiding in counteracting intrinsic radiation resistance effects (*e.g.* from p53 mutations).^8^ Similar effects have been observed with, *e.g.*, silver^151,152^ and titania NPs.^153^ However, none of these studies were specifically conducted with a focus on intrinsic radiosensitivity. There are only few studies available correlating the nanoparticle radioenhancement efficiency to the intrinsic radiosensitivity of cell lines. Marill *et al.* (2014) found a positive correlation between dose enhancement of HfO_2_ (NBTXR3) NPs and the intrinsic radiosensitivity of different cancer cell lines *in vitro*.^24^ The underlying mechanisms remain unknown. At this stage and taking our lack of mechanistic understanding into consideration, it will be more important to identify optimal NPs with a selective advantage for general tumor cell *versus* normal tissue radiosensitization than the design of NPs directed for specific genetic tumor cell make-ups.

#### Reactivation of an antitumor immune response

On the clinical level, the recent achievements in imaging technologies and highly conform radiotherapy have resulted in a shift from classic low-dose fractionated radiotherapy regimens to hypofractionated and stereotactic body radiotherapy applying single or only a few high dose radiotherapy fractions to individual tumor sites. While previously thought to be immunosuppressive, recent studies have clearly demonstrated an important relationship between hypofractionated radiotherapy and the immune system. Its exploitation can achieve impressive responses, which have not been observed by conventional fractionated radiotherapy. Indeed, high doses of irradiation has been shown to instigate potent immune responses, and has been classified as an ‘*in situ* vaccine’, whereby the induction of immunogenic cell death (ICD) sets off an inflammatory cascade. This results in the release of antigens, damage-associated molecular pattern (DAMP) molecules and type 1 interferons, activating antigen presenting cells such as dendritic cells (DCs) in the tumor. In turn, DCs upregulate co-stimulatory molecules, which facilitate successful priming of CD8^+^ T cells, ultimately resulting in the recruitment of antigen specific immune cells to the tumor. While in some cases this leads to an effective and efficient anti-tumor response,^154^ radiotherapy alone is often not sufficient enough to overcome the immunosuppressive tumor microenvironment (TME), and as such the potency of infiltrating immune cells is often hampered. A combined treatment modality of NPs and radiotherapy does result in enhanced direct cytotoxicity and tumor control mediated *via* the multiple mechanisms related to the first *R*s of radiotherapy. Eventually increased amounts of small DNA fragments, antigen presentation, and release of DAMPs may also stimulate immunogenic cell death as demonstrated for Au^155^ and HfO_2_ NPs^156^ and might even lead to a systemic immune response towards abscopal effects alone and in combination with immune response stimulatory agents.^157,158^ In a study with anti-PD1–resistant tumor model mice, HfO_2_ nanoparticles in combination with radiotherapy and anti-PD1 produced abscopal effects.^157^ In another study, the combination of HfO_2_ nanoparticles with radiotherapy and NF-αCTLA4 and αPD1 check point inhibitors improved the control of local tumors and metastases.^159^ However, at this stage, we have insufficient insights how to rationally direct these processes with NPs towards immunogenic cell death and an increased therapeutic window.

The emerging understanding of the underlying biological mechanisms for nanoparticle-based radioenhancement is important for the design of safe and effective nanomedicines. However, studies focusing on materials other than gold are scarce. Further (comparative) investigations into relevant materials including hafnium, titanium, iron, gadolinium, and silver metals are required to understand how these particles interact with biological matter in the context of radiation biology. As has been pointed out previously, the nanotoxicology field might be an underutilized resource in this regard, and collaborative efforts with this field should be established and reinforced.

##### Quantifying biological mechanisms contributing to damage

A wide range of methods has been established to study the biological effects of nanoparticles. Specific, often well-established, assays linked to the 6 *R*s may be used. Examples include the comet^160^ and γ-H2AX^117,161^ assay to address DNA damage and repair, the TUNEL^162,163^ assay and micronuclei counting to assess apoptosis and quantify mitotic catastrophe, respectively, or fluorophores such as H2DCF-DA to assess *in vitro* ROS production. In addition, gene and protein expression studies may provide a powerful tool to identify treatment-induced signaling processes and to improve our mechanistic understanding. A common and effective approach to investigating the overall radiation response *in vitro* is the clonogenic cell survival (or colony formation) assay, established over 60 years ago and is considered to be the gold standard to quantify^164^ the cells’ reproductive abilities in response to irradiation. However, the clonogenic cell survival assay is tedious and time-consuming and offers very low sample throughput.^165,166^ As such, metabolic-based assays (*e.g.* MTT or CellTiter-Glo®) can in part overcome these limitations and are often used as a first screening approach to identify reasonable NP concentration and ionizing radiation dose ranges to probe novel combined treatment modalities on the *in vitro* level, followed by the more exact clonogenic cell survival assay.^94,165,167^ See, for example, Subiel *et al.* (2016)^168^ for a detailed review on radiobiological techniques and their quantification *in vitro*. Eventually, the combined treatment modality will have to be probed in respective *in vivo* tumor models (subcutaneous *vs.* orthotopic tumors; immuno-compromised *versus* immuno-competent hosts) in order to evaluate NP-mediated radioenhancement towards an enlarged therapeutic window.

## 4. Nanoparticles candidate materials with radioenhancing properties

**Fig. 3:**
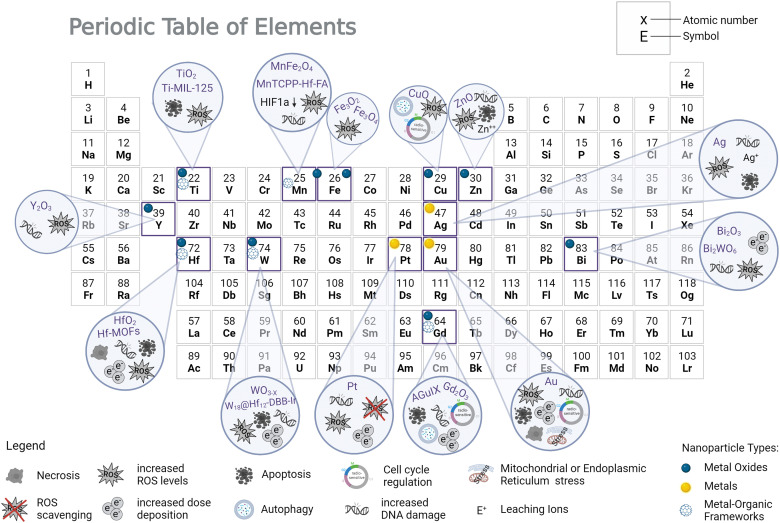
Overview of major elements applied as radioenhancer candidate materials and suggested mechanisms of action. Elements may be applied in nanoparticulate form as metal, metal oxide or metal organic framework, or in ionic form.

Early findings of using substances to enhance radiation damage by means of the photoelectric effect go back to the 1980s, where iodine contrast medium was found to sensitize cells to X-rays.^169,170^ Before that, it was already well known, that the dose absorbed by tissue at the boundary to a higher-*Z* material (such as bone) is greatly enhanced.^171,172^ Around two decades ago, gold metal foil or microspheres have been shown to enhance the cytotoxic effect of ionizing radiation due to the release of secondary radiation using kV X-rays.^173,174^ Using platinum-DNA complexes, Kobayashi *et al.* were able to conclude from their experiments, that platinum atoms acted as enhancers of X-ray-induced DNA breaks by increasing the production of hydroxyl radicals due to photoelectric and Auger effects.^175^ Until now, several metals and metal oxides have been investigated as NP radioenhancers, either *in silico*, *in vitro*, or *in vivo*, with Au NPs being the most extensively studied formulation in literature.^1,67^ They appear to be an intuitive choice as they possess seemingly good biocompatibility, passive accumulation in tumors, low toxicity, and easy synthesis methods.^25,38^ Most importantly, their atomic number is very high (*Z* = 79), which is particularly relevant for kV photon interactions. Nevertheless several studies indicated a decreased, yet appreciable gold nanoparticle radioenhancement effect at MV *vs.* kV X-ray treatment.^109,176^ Hafnium-based NPs have already completed clinical trials, and gadolinium-based NPs have entered trials; both display good dose enhancement effects under X-ray irradiation.^4,24,177,178^ In addition, various other metal-related materials have proven to be effective enhancers of X-rays, such as materials with silver, platinum, titanium, tungsten, iron (SPIONS), zinc, or bismuth metals (Table 1 and Fig. 3). All materials listed in Table 1 show promising therapeutic efficiency *in vitro* and *in vivo* for kV or clinically relevant MV irradiation. Radioenhancement success is mostly described by increased DNA damage, ROS levels, and apoptosis levels. While chemical and physical interaction of nanoparticles and irradiations are easily accessed and well described, in-depth biological response mechanisms are investigated only in some cases. While the therapeutic performance of single nanoparticles can be tailored by size or surface functionalization, hybrid materials offer the integration of multimodal imaging and/or multimodal therapy options.

**Table 1:** Studies on single or hybrid nanoparticle (NP) formulations

NP type	Model	Beam sources	Important outcomes	Ref.
**Single metal and metal oxide nanoparticles**				
TiO_2_ (un-/doped)	PRESAGE phantom	kV Photons	• X-ray energy differential therapeutic effects observed in PRESAGE	94 and 182–184
	*In vitro*	MV photons	• NP radioenhancement effect during MV X-ray irradiation explained by increased ROS generation	
	*In vivo*		• Rare earth dopants (*e.g.* Sm, Gd, Nd, Eu, Er, Tb) increased X-ray induced ROS, and apoptosis markers	

(PAA-) TiO_2_/H_2_O_2_	*In vitro*	kV Photons	• NPs increased intracellular H_2_O_2_ concentrations by gradual release thereof leading to increased radiotherapy efficiency	163, 185 and 186
	*In vivo*		• Increased ˙OH radical and H_2_O_2_ concentration, DNA damage and apoptosis during X-ray irradiation	

MnFe_2_O_4_	*In vitro*	kV Photons	• Oxygen delivery *via* catalytic H_2_O_2_ decomposition	144 and 187
	*In vivo* (normoxia and hypoxia)	MV photons	• Hypoxic conditions were alleviated *in vitro* and *in vivo*	
			• Decreased HIF-1a levels, and increased apoptosis and DNA damage under irradiation during hypoxic conditions	
			• Immune-modulating effect: suppression of PD-L1 expression and increased infiltration of T cells even after irradiation	
			• PEGylated NPs showed good cytocompatibility, passive tumor accumulation, O_2_ generation *via* H_2_O_2_ decomposition, GSH consumption *via* glutathione-peroxidase-like activity, enhanced ROS and double-strand break levels in hypoxic conditions, and hypoxia attenuation and good radioenhancement efficiency *in vivo*.	

SPIONS (γFe_2_O_3_, Fe_3_O_4_)	*In vitro*	kV Photons	• Excellent biocompatibility	81
			• Increased ROS production *via* Fenton and Haber–Weiss reactions from released iron ions and catalytically active nanoparticle surface	
			• X-ray irradiation led to additional oxidative stress from increased catalytically active nanoparticle surfaces	

CuO	*In vitro*	MV photons	• Increased ROS levels with X-ray treatment and CuO NPs	83
	*In vivo*		• Increased radiosensitivity by the NP-induced modulation of the cell cycle distribution towards increased G2/M phase	
			• Increased level of self-destructive autophagy observed with the combination of CuO NPs and X-rays	

ZnO	*In vitro*	kV Photons	• Radioenhancing effects explained by increased apoptosis and DNA damage; oxidative stress as possible driver identified	188–191
	*In vivo*	MV photons	• Discussed cytotoxic effects of ZnO: dissolution of Zn^++^ in acidic conditions; e^−^h^+^ pair production even in the dark leading to surface ROS production	
			• Discussed genotoxic effects of ZnO: oxidative stress	
			• Gd-doped ZnO NPs increased cells in G1 phase and decreased DNA repair efficiency	
			• ZnO-CaffeicAcid NPs showed radioenhancement *via* ROS/oxidative stress generation, DNA damage, DNA repair, and mitochondrial dysfunction, suppression of cell cycle checkpoint machinery and cell death promotion (*via* apoptosis, necrosis, and disregulations of gene and protein expressions)	

Y_2_O_3_	*In vitro*	kV Photons	• Increased ROS and DNA double-strand breaks with NPs alone or in combination with X-rays	103
			• NPs affected irradiation induced DNA damage and repair response	
			• Synergistic effects of NP treatment and irradiation proposed by clonogenic assay	

Ag	*In vitro*	MV photons	• Increased apoptosis levels after irradiation with Ag NPs	192–197
	*In vivo*		• Antiproliferative activity in combination with radiotherapy	
			• Decrease of mitochondria membrane potential, promotion of apoptosis and enhanced destructive autophagy under irradiation in hypoxic conditions with Ag NPs	
			• Increased ROS and protective autophagy during irradiation with Ag NPs	
			• Radiosensitization might be Ag^+^ cation release dependent	

Gd chelate (AGuIX)	*In vitro*	kV Photons,	• Good safety profile	2, 178 and 198–202
	*In vivo*	In human	• Rapid and safe renal elimination	
		MV photons	• Preferential accumulation in tumor due to EPR effect after intravenous injection	
		MeV hadrons	• Possible emission of low-energy photoelectrons and Auger electrons leading to higher ROS	
			• Dose enhancement higher for kV than MV photon, and C^6+^ than He^2+^ ion irradiation	
			• Photon radiation-induced ROS and DNA double-strand break increase and DNA repair reduction observed	
			• Ion irradiation increased DNA damage mediated by ROS	

Gd_2_O_3_	*In vitro*	kV Photons	• Compared to a solution of separate Gd-atom species, Gd_2_O_3_ NPs showed higher ROS generation, suggesting a Gd–Gd interatomic de-excitation-driven nanoradiator effect	203 and 204
		MeV protons	• Increased hydroxyl radical and ROS production, increased DNA double-strand breaks and cell cycle arrest at G2/M phase	
		C^6+^ ions	• Increased apoptosis and cytostatic autophagy identified as radiosensitization mechanism	

HfO_2_ (NBTXR3)	*In vitro*	kV Photons	• NBTXR3 has a good safety profile and improves radiotherapy efficiency *via* physical mechanisms	4, 24, 156–159 and 205–208
	*In vivo*	In human	• Increased necrosis, DNA double-strand breaks, micronuclei formation and an activation of the cGAS-STING pathway detected after irradiation	
		MV photons	• In combination with radiotherapy: enhancement of early apoptosis, early necrosis and late apoptosis/necrosis; abscopal effects driven by an increased CD8+ cell infiltration	
			• Modulation of the immunopeptidome observed *in vivo* leading to an anti-tumor immune response	

WO_3−*x*_	*In vitro*	kV Photons	• Increased DNA double-strand breaks and apoptosis after irradiation with NPs	209 and 210
	*In vivo*		• Remarkable synergistic effect of radiotherapy and phototherapy (PTT/PDT)	

Pt	*In vitro*	kV Photons	• ROS scavenging capabilities of Pt NPs during irradiation detected, which might counteract nanoparticle radioenhancement	70 and 211–213
		MV photons	• Pt NPs amplified radiation therapy by confined production of ROS in nano-volumes around nanoparticles	
		C^6+^ ions	• Amplified DNA damage detected during hadron therapy	

Au	*In vitro*	kV Photons,	• Physical, chemical and biological mechanisms described	1, 45, 104, 109, 121, 124, 176 and 214–220
	*In vivo*	MV photons,	• Less radioenhancement observed at MV compared to kV irradiations	
		Protons	• Observed enhancement effects at MV energies cannot be explained by physical effects	
			• Enhancement effects are cell-line specific	
			• Cell cycle arrest in G2/M phase has been observed for some cell lines	
			• Cytoplasmic damage can drive DNA damage; mitochondrial function identified as possible driver	
			• Increased DNA dmage and/or mitochondrial and/or ER stress with and/or without irradiation lead to increased apoptosis and/or necrosis	
			• Au NPs have radiosensitization effect *via* various biological mechanisms, such as weakening the detoxification system	

Bi_2_O_3_	*In vitro*	kV Photons	• *In vitro* enhancement ratio for kV irradiation higher compared to MV irradiation	62, 66 and 221
		MV photons	• Monte Carlo studies predicted dose enhancement for kV irradiation but not for MV irradiation	
			• Radiocatalytic surface of NPs hypothesized to lead to water splitting	

BiFeO_3_	Gel dosimetry	kV Photons	• Promising results in radiotherapy amplification, magnetic hyperthermia and imaging	222
	*In vitro*			

BiGdO_3_	MAGIC gel dosimeter	kV Photons	• Multifunctional pegylated nanoparticles showed radiation enhancement in gel dosimetry, *in vitro* and *in vivo*.	223
	*In vitro*			
	*In vivo*			

Bi_2_WO_6_	*In vitro*	kV Photons	• Bi_2_WO_6_ generated radiocatalytic ROS in an acellular system, with ˙OH as main oxidative species identified	224
	*In vivo*	MV photons	• Radiosensitization *in vitro* was found in a clonogenic assay together with increased ROS and DNA double strand break levels	
			• Efficient radiotherapy enhancement shown *in vivo* without toxicity effects in the time span of 30 days.	

**Hybrid nanoparticle systems**				
Au–TiO_2_ hybrid	*In vitro*	kV Photons	• Production of large amount of ROS during irradiation	225
	*In vivo*		• Synergistic X-ray therapeutic effect of Au and TiO_2_ due to their interfacial contact	

Au@MnO_2_-PEG	*In vitro*	kV Photons	• O_2_ generation from H_2_O_2_ decomposition *via* MnO_2_ shell and relieve of cellular hypoxia	226
	*In vivo*		• H_2_O_2_ decomposition led to Mn^2+^ release and enhanced *T*_1_-weighted MR imaging	
			• Compared to the individual components, Au@MnO_2_ core@shell hybrid NPs led to synergistic radioenhancement effects *in vitro* and *in vivo* and to more increased double-strand breaks and apoptosis levels	

Au@Pt nano-dendrites	*In vitro*	Photons (energy N/A)	• Integration of CT imaging, PTT & RT with synergistic therapeutic effects	227

Au:Pt-PEG	*Plasmid DNA*	kV Photons	• NPs enhanced the MV X-ray induced double-strand breaks in plasmid DNA mainly by influencing the chemical stage that takes place after the physical interaction	228 and 229
	*In vitro*	MV photons	• NP radioenhancing effect with kV X-rays observed *in vitro* and *in vivo* by enhancing double-strand breaks, apoptosis and relieving of hypoxia *via* H_2_O_2_ decomposition	
	*In vivo*			

WS_2_:Gd^3+^-PEG 2D-nanoflakes	*In vitro*	Photons (energy N/A)	• Triple-modal imaging: CT/MR/photo acoustic	230
	*In vivo*		• Synergistic PTT/RT therapeutic effects observed	
			• X-ray enhanced DNA double-strand breaks observed	

Gd_2_(WO_4_)_3_:10%Tb@PEG/MC540	*In vitro*	kV Photons	• X-ray induced generation of ^1^O_2_ observed from photo excitation of surface coupled merocyanine (MC) 540	231
	*In vivo*		• Synergistic radioenhancement and PDT effects observed *in vitro* and *in vivo*	
			• Dual-modal imaging properties (CT/MR)	

Fe_3_O_4_@Ag	*In vitro*	MV photons	• Synergistic radioenhancement effects of the Fe_3_O_4_ core and Ag shell as compared to the individual components alone	232
			• Radiosensitivity enhancement through decrease of the cytoprotective autophagy at an early stage, followed by an increase of calcium-dependent apoptosis at a later stage	

Gd_2_O_3_/BSA@MoS_2_-HA	*In vitro*	kV Photons	• Enhanced DNA double-strand breaks in combination with X-rays *in vitro*	233
	*In vivo*		• *In vivo* tumor growth inhibition in combination with X-rays	
			• Best therapeutic effects in combination with X-ray and PTT treatments	
			• *In vivo* multimodal imaging properties (MSOT/CT/MR)	

BiNPs@SiO_2_@BamCS/PCM	*In vitro*	kV Photons	• Acellular detection of elevated ROS (˙OH, ^1^O_2_ and ˙O_2_^−^) under X-ray irradiation	234
	*In vivo*		• Elevated cell death, ROS and DNA double-strand breaks detected in combination with X-rays *in vitro*	
			• Depleted GSH levels due to hyperthermia-released and H_2_O_2_-activated proalkylation agent BamCS	
			• Excessive ROS and irreversible depletion of endocellular GSH led to cell death *via* mitochondria-mediated apoptosis pathway	
			• Tumor growth inhibition in combination with X-rays	
			• Synergistic PTT/RT effects observed	

**Nanoscale metal–organic frameworks (nMOFs)**				
MnTCPP–Hf–FA	*In vitro*	kV Photons	• O_2_ generation *via* catalytic H_2_O_2_ decomposition	235
	*In vivo*		• Decreased HIF-1a expression suggested hypoxia alleviation due to intracellular H_2_O_2_ decomposition and O_2_ generation	
			• Increased ROS levels and DNA double-strand breaks after irradiation even in hypoxic conditions	

UiO-66-NH_2_(Hf)	*In vitro*	Photons (energy N/A)	• Radioenhancement due to increased ROS levels, DNA double-strand breaks and cell apoptosis	236
	*In vivo*			

Hf_6_-DBB-Ru	*In vitro*	kV Photons	• MOFs accumulated in mitochondria through cationic nature of Ru-based photosensitizer	237
	*In vivo*		• Irradiation induced ˙OH radical generation from Hf_6_ units and ^1^O_2_ generation from the DBB-Ru photosensitizer	
			• Clonogenic radioenhancement effects observed, along with increased double-strand break, ^1^O_2_, lipid peroxidation (COX-2 upregulation) and apoptosis/necrosis levels after irradiation	
			• Irradiation induced depolarization of the mitochondrial membrane leading to cytochrome *c* release and caspase-3 activation (apoptosis pathway)	
			• Effective *in vivo* tumor regression after intratumoral or intravenous nMOF administration and low dose (6 or 8 Gy) X-ray therapy, with increased levels of necrosis and apoptosis.	

W_18_@Hf_12_-DBB-Ir	*In vitro*	kV Photons	• Hierarchical assembly of Hf_12_, DBB-Ir, and W_18_ generated 3 distinct ROSs: ˙OH, ^1^O_2_ and ˙O_2_^−^, respectively	238
	*In vivo*		• Irradiation *in vitro* lead to enhanced ROS, DNA double-strand breaks and apoptosis/necrosis	
			• Superb anticancer efficacy (>99% tumor growth inhibition) on two *in vivo* tumor models with irradiation	

To understand which materials are most efficient in radiotherapeutic settings, material comparison studies (in clinically relevant settings) are highly valuable. Table 2 lists a selection of such studies. While in some studies the radio- and ROS-enhancing efficiency scaled with the atomic number,^53^ other comparison studies showed less atomic number dependency. For example, Ag NPs have outperformed Au NPs due to higher biological radiosensitization mechanisms.^179^ A recent investigation by Guerreiro *et al.* found that out of 22 metal oxide NPs examined, TiO_2_ and V_2_O_5_ showed the most significant damage to plasmid DNA probes and increase in ROS generation under 6 MV X-ray irradiation. This draws attention to the importance of physico-chemical surface effects and ROS generation at clinically relevant MeV X-ray energies and highlights the role of photocatalysts (especially TiO_2_) in radioenhancement.^96^ These findings also direct attention away from high-*Z* nanoparticles as clinically relevant radioenhancers and direct it towards photocatalysts, which can also be low-Z nanoparticles. Recent comparisons of low- to high-*Z* nanomaterials under kV and MV X-rays and under MeV proton irradiation also demonstrated these trends.^55^ Additionally, metal organic frameworks (MOFs) with highly accessible surface area have been shown to outperform their metal oxide nanoparticle counterpart in ROS generation and radiotherapy efficiency and can even be loaded with therapeutic drugs.^101^

**Table 2:** Studies comparing multiple types of nanoparticle (NP) radioenhancers

NP type	Model	Beam sources	Important outcomes	Ref.
Nanoparticle comparisons				
Ag and Au NPs	*In vitro*	MV photons	• Ag NPs showed higher radiosensitizing ability compared to Au NPs, combined with increased apoptosis and authophagy levels	179
	*In vivo*			

Pt and Gd-based (AGuIX) NPs	Plasmid DNA	MeV protons	• Pronounced nanosize damage (>2 nm) at the end of proton track (Bragg peak)	40, 163, 185 and 186
			• Gd-based NPs less capable of producing complex lesions than Pt NPs	
			• DMSO (radical scavenger) reduced plasmid damage, hydroxyl radicals identified as important mediators for both NPs	

HfO_2_ NPs, Hf_6_- & Hf_12_-DBA MOFs	APF acellular assay	kV Photons	• MOFs generated more ˙OH radicals compared to HfO_2_ at same Hf molar concentration	101
	*In vitro*	Co60 photons	• Better local radiotherapy outcomes for MOFs than HfO_2_	
	*In vivo*		• MOFs in combination with PD-L1 checkpoint blockade induced systemic antitumor immunity	

Au NPs, SPIONS, PtNDs (Pt nano dendrites), BiNRs (Bi_2_O_3_ nano rods)	*In vitro*	MeV protons	• Proton beam irradiation with nanoparticles enhance ROS creation	53
			• ROS generation and *in vitro* radioenhancement biggest for BiNRs > PtNDs > Au NPs > SPIONS	

Comparison of 22 metal oxides	Plasmid DNA	MV photons	• Surface chemistry of NP important criterion for success	96
	Aequous ROS (˙OH, ˙O_2_^−^, ^1^O_2_) probes		• Only TiO_2_ and V_2_O_5_ showed ROS and DNA damage enhancement and were identified as good radiosensitizers, with V_2_O_5_ having a too high toxicity profile	

PAA-TiO_*x*_ NPs and Au NPs	Aequous ROS probe	kV Photons	• H_2_O_2_ identified as important mediator of the more effective and generally safe PAA-TiO_*x*_ NP radioenhancer (Au NPs were tested at significantly lower mass concentrations)	239
	*In vitro*			
	*In vivo*			

SiO_2_, TiO_2_, TiN, ZrO_2_, WO_3_, HfO_2_, Au	Aequous ROS (H2DCF-DA) probe	kV Photons	• Dose enhancement efficiency for kV photons follows physical high-*Z* rationale	55
	*In vitro*	MV photons	• Surface catalytic properties/ROS enhancement important for MV photons and protons	
		MeV protons	• TiO_2_, WO_3_ performed best in ROS production under all radiation sources	
			• Dissolution of WO_3_ led to limited radioenhancment	

Au and SPION-DX (Dextran coated iron oxide) NPs	*In vitro*	MV photons	• Enhancement effects were cell-line differential	240
			• At similar metal-mass uptake Au NPs showed higher enhancement than SPION-DX	

MOFs: Hf-DBA, Hf-TCPP, Ti/Zr-PCN-415, Ti-MIL-125	*In vitro*	kV Photons	• Greatest *in vitro* radio-enhancement effects were found for Ti-MIL-125, followed by Ti/Zr-PCN-415 nanoMOFs	241
Oxide NPs: HfO_2_, ZrO_2_, TiO_2_			• nanoMOFs outperformed corresponding equi-molar metal oxide nanoparticles in *in vitro* X-ray radio-enhancement	

Nanoparticle radioenhancement studies using proton therapy are less widely available. While the effects of gold NP size, coating, and beam energies under proton irradiation have been investigated extensively through Monte Carlo simulations,^38,180^ only few materials have been tested *in vitro* or *in vivo* with proton irradiation, with the main focus so far on gold, platinum, gadolinium and iron-related materials.^40,45^ It is perceived that for proton therapy, chemical and biological mechanisms are more relevant than physical mechanisms.^181^ In view of the pivotal role proton therapy plays for certain patient groups and the ongoing global expansion and advancement of this treatment modality, efforts should be made to enhance the understanding of proton-NP interactions.

## 5. Nanoparticle-aided augmentation of other radiation therapy techniques

In addition to a combined treatment modality of NPs with external beam radiotherapy, NPs can also be used to enhance the efficacy of other radiation-based cancer treatment modalities, such as brachytherapy or phototherapy. In brachytherapy, radioactive seeds are locally implanted into the tumor tissue and it is therefore also called internal radiation therapy. In combination with kV photon-emitting radioseeds, high-*Z* nanoparticles can be applied to increase the dose deposition, leveraging the photoelectric effect. Another way of performing brachytherapy is by the incorporation of radioactive elements (such as α, β, or Auger electron emitters) into nanosized agents (such as Au or iron oxide NPs). This technique is called nanobrachytherapy or nanoseed brachytherapy and has made advancements in preclinical research to overcome limitations of traditional millimeter-sized radioactive seeds, such as inhomogeneous dose deposition and technical applications.^242^ For instance, intratumoral injections of Au NPs labeled with ^177^Lu (β emitter), ^225^Ac, or ^211^At (α emitters) have shown good growth inhibition of breast, glioma, or pancreatic tumors in rodents.^243–245^ The choice of the radioactive element depends on the therapeutic suitability and involves considering several properties such as decay type, half-life, tissue penetration depth, LET, and toxicity of the parent and daughter element.^242^ The choice of the radiolabeled nanoparticle also depends on therapeutic and practical factors, such as labeling technique (chelator-based or chelator-free), its ability for surface functionalization, biocompatibility/toxicity, and imaging or multifunctional capability.^242^ Since most of the radionuclide-emitted particles are in the keV energy range, it is still a matter of debate whether or not an additional amplification *via* the radioenhancement effects of high-*Z* nanoparticles can be expected from internal brachytherapy sources. An *in silico* investigation estimated a radioenhancement effect of up to 20% from iron oxide nanoparticles and different clinically used radionuclides depending on the isotope proximity and nanoparticle clustering distance.^246^ Similarly, it has been shown *via* Monte Carlo simulation,^247^*in vitro*,^248^ and *in vivo*^249^ experiments that the radioenhancement properties of Au nanoparticles can be leveraged for internal brachytherapy.

Nanoparticles can also be used for (solid) cancer treatment *via* phototherapy, which uses photon sources of much lower energy (UV to NIR) compared to X-rays. The two most important modalities are photothermal therapy (PTT), in which photons are converted into toxic heat, and photodynamic therapy (PDT), in which photons are converted into the production of ROS.^250^ For NP-based PTT, nanoparticles ideally should have a high absorption and photothermal conversion efficiency in the NIR-II range (1000–1350 nm) paired with a high biocompatibility and low toxicity.^251^ Most inorganic NPs create heat *via* surface plasmon oscillations (also called LSPR, or localized surface plasmon resonance), while organic PTT agents transfer heat *via* vibrational relaxation upon photoexcitation.^251^ Nanoparticles turning NIR light into heat that have been used as synergistic PTT and radioenhancing agents, include WO_3−*x*_, Au@Pt dendrites, WS_2_:Gd^3+^-PEG 2D-nanoflakes, Gd_2_O_3_/BSA@MoS_2_-HA and BiNPs@SiO_2_@BamCS/PCM (Table 1). For NP-based PDT, fluorescent dye photosensitizers (PSs) are supplied *via* nanoparticles to the region of interest, where they transfer energy from incoming photons to surrounding oxygen molecules to generate toxic ROS, typically ^1^O_2_.^252^ Most PS molecules alone, such as porphyrins or chlorins, are hydrophobic and prone to aggregate intracellularly, which imposes a quenched fluorescence emission as well as reduced singlet oxygen generation.^253,254^ While one strategy to overcome this challenge is by developing fluorogens with aggregation induced emission (AIE) characteristics, such as TPECM-2TPP,^254^ such molecules rely on low-energy excitation wavelengths from laser light sources for efficient excitation. Therefore, nanoparticles for PDT play roles either as a carrier or as an absorber of incoming light that activates the surrounding PS molecule, which has an intrinsic absorbance range. Since UV, visible, and NIR light have low tissue penetration depth, X-ray-triggered PDT has been used to treat deep-seated tumors. X-rays can be converted into a form of light that can excite PSs *via* light-scintillating nanoparticles (*e.g.* rare-earth based nanoparticles).^255^ X-ray-triggered PDT has been proven to be successful *in vivo*, using, for example, radioenhancing Gd_2_(WO_4_)_3_:Tb nanoparticles as scintillator and merocyanine 540 as PS (Table 1),^231^ or a metal doped silicate nanoscintillator with rose bengal as PS.^256^ Photosensitizers can also be integrated into MOFs to additionally produce ^1^O_2_ next to ˙OH upon X-ray exposure, as shown for Hf_6_-DBB-Ru or W_18_@Hf_12_DBB-Ir (Table 1).^237,238^ A rather new type of PDT agent is copper-cysteamine (Cu-Cy) nanoparticles, which can produce ROS, including ^1^O_2_, directly under UV light, X-rays, microwaves, or ultrasound, and has shown *in vitro* and *in vivo* therapeutic efficacy.^257–259^

## 6. Clinical progress

Gold nanoparticles remain the best investigated nanomaterial for the enhancement of radiotherapy, with first preclinical success already achieved in 2004.^1^ To our knowledge, no gold formulation is being evaluated in combination with radiotherapy in clinical studies. The main challenge of gold nanoparticles that prohibits their translation into clinical usage is the incomplete understanding of their biological fate, safety, and long-term biocompatibility *in vivo* with respect to its physico-chemical properties.^8,260,261^ Although not developed as radiosensitizers, a few gold nanoformulations have overcome the toxicity problem and transitioned into clinical studies, including materials such as CYT-6091 (Cytimmune Sciences, USA) or AuroShells® of the AuroLase™ therapy (Nanospectra Biosciences, USA).^8^ The latter therapy aims to ablate solid tumors using near infrared light, while CYT-6091 is a drug delivery gold nanoparticle with bound tumor necrosis factor-α on its surface. The successful clinical transition of these particles raises the hope for gold nanoformulations also entering clinics as radiosensitizers.

Hafnium dioxide-based radioenhancer nanoparticles (NBTXR3) developed by Nanobiotix are currently under evaluation in several clinical trials, both as a single agent added to radiotherapy or in combination with chemo- or immunotherapeutic agents. Studies with NBTXR3 as a single agent involve the radio-treatment of lung (Phase I, NCT04505267), pancreatic (Phase I: NCT04484909), liver (completed Phase I: NCT02721056), head and neck (Phase III: NCT04892173, Phase I: NCT01946867), and soft tissue (completed Phase III: NCT02379845) cancers. Studies with NBTXR3 that include radiation and immunotherapy are under investigation for the treatment of recurrent head and neck, lung, or liver metastasis (Phase I: NCT03589339, drugs: Nivolumab, Pembrolizumab), head and neck cancers (Phase II: NCT04834349, drug: Pembrolizumab; Phase III: NCT04892173, drug: Cetuximab), or solid tumors (Phase I/II: NCT05039632, drugs: Ipililumab, Nivolumab). Combinational studies of NBTXR3 with radiation and chemoterapeutic drugs are clinically investigated for the treatment of esophageal (Phase I: NCT04615013, chemotherapeutic drugs: capecitabine, carboplatin, docetaxel, fluorouracil, leucovorin, oxaliplatin, paclitaxel) and head and neck (Phase II: NCT04862455, chemotherapeutic agent: pembrolizumab) cancers. Positive completion of phase III clinical trials for the treatment of locally advanced soft-tissue sarcoma, and the subsequent CE Mark approval of NBTXR3 (under the name of Hensify®) in 2019, represents the first demonstration of a radioenhancer to provide therapeutic benefits in synergy with standard radiotherapy treatment methods.^207,262^

A second radiotherapy-enhancing formulation being evaluated in clinical trials in combination with radiotherapy is a polysiloxane Gd-chelate-based nanoparticle called AGuIX. Current studies investigating the use of AGuIX as a radiosensitizer with radiation alone are focusing on the treatment for brain metastasis (completed Phase I: NCT02820454, Phase II: NCT03818386, NCT04899908) and for lung tumors and pancreatic cancer (Phase I/II: NCT04789486). AGuIX is also evaluated in combination with a chemotherapy drug (Temozolomide) for the radiotreatment of glioblastoma (Phase I/II: NCT04881032) and in combination with chemotherapy (cisplatin) and brachytherapy for the radiotherapy treatment of gynecologic cancers (Phase I: NCT03308604). As a first radiosensitizer, AGuIX is also enrolled for a phase II study for proton therapy of recurrent tumors (NCT04784221). Attempts have recently been made to further optimize AGuIX particles by adding the feature of copper chelation (CuPRiX) with the aim of reestablishing copper homeostasis.^263^

Another emerging radioenhancement formulation is rare-earth-doped TiO_2_ nanoparticles (Oxilia), which increase ROS during radiotherapy through water splitting. The company Xerion Healthcare, based in the UK, has shown promising results in the pre-clinical stage in *in vitro* and *in vivo* mouse xenograft models, claiming effectiveness of their nanoparticle formulation in pancreatic cancer models.^264,265^ In 2019, further funding was secured to support clinical investigations, none of which have been initiated to date. It will be interesting to follow the further development and clinical translation of these known photocatalysts as radioenhancers and how they perform in clinical trials in comparison to, *e.g.* hafnia-based particles.

A few iron formulations are in clinical trials for various cancer therapies.^266^ Although developed and FDA-approved for the treatment of iron deficiency anemia, Ferumoxytol (iron oxide NPs) may serve as a radiotherapy adjuvant, releasing iron during irradiation.^267^ Ferumoxytol is now in clinical trials for the therapy of primary and metastatic hepatic cancers (NCT04682847). Though not developed as a radioenhancer, another iron oxide nanoparticle formulation, NanoTherm® (developed by MagForce) is FDA approved and currently in clinical trials for the focal ablation of prostate cancers using a magnetic field (Phase IIb: NCT05010759). Thus, iron oxide might in the future combine and enhance two cancer treatment strategies, hyperthermia and radiotherapy, that possess synergistic therapy potential.^268^

From the new classes of materials, a MOF formulation, RiMO-301, is now in clinical trials for the radiotherapy of advanced tumors (Phase I: NCT03444714).

The few nanoparticle formulations which are being evaluated in clinical trials are not reflecting the plethora of nanoparticles which have been tested preclinically (see *e.g.*Tables 1 and 2). The translation of a nanoparticulate material from bench to bedside is a long process with the safety being a key priority. Relevant barriers for the clinical translation of nanoparticle radioenhancers have been identified by a dedicated multi-disciplinary cooperative in 2018.^7^ Amongst them were (i) the discovery of radioenhancement mechanisms, including the standardization of experimental methods to allow meaningful comparison of nanoparticle systems, (ii) the *in vivo* fate of nanoparticles, (iii) understanding patient priorities, as well as (iv) the nanoparticle manufacturing and its scalability, which should ideally be integrated into the early nanoparticle design phases. While the knowledge on topics (i)–(iii) is constantly updated by ongoing research activities, it is interesting to note that most, if not all, of the above-mentioned clinical formulations are synthesized by wet-chemical, batch synthesis methods, for which upscaling can pose a big challenge, especially when high nanoparticle quantities become necessary for clinical evaluations or after regulatory product acceptance. Nanoparticle production *via* continuous flow processing methods, including flame spray pyrolysis, can offer production rates on lab and industrial scales (mg to kg per day), while offering significant versatility in nanoparticle design in a one-step process.^269–271^ Testing those materials already in pre-clinical stages could accelerate nanoparticles entering clinical translation.

## 7. Delivery of radioenhancers

The targeted delivery of nanoparticles to tumors remains a major focus of research. Based on the recent meta-analysis by Wilhelm *et al.*,^272^ an average of less than 1% of the injected dose of nanoparticles typically accumulates in the tumor tissue, irrespective of active or passive delivery approaches. Interestingly, the tumor accumulation can be increased to around 10% by kinetically saturating the uptake of the liver by high dose injections of inactive nanoparticles, however, off-target accumulation is still considerable.^273^ For radioenhancement, therapeutically effective nanomaterial concentrations in the tumor are typically rather high, and are therefore especially challenging to achieve by conventional intravenous administration. Therefore, current clinical studies (both for HfO_2_ (Hensify)^274^ and for nanoparticle tumor hyperthermia)^275^ employ intratumoral injection as a preferred route of delivery. While clinically successful (improved patient outcome), this delivery route limits the applicability of the therapy to a small subset of patients with only locally advanced and well-accessible tumors. Alternative delivery strategies to tumors, and their effect on outcome, are therefore a major priority in the field for increasing the impact by making nanoparticle radioenhancement accessible to a larger cancer patient population.

## 8. Conclusions and future directions

Overall, a plethora of radioenhancer material candidates have been tested and evaluated in numerous experimental studies, showing excellent therapeutic anticancer efficacies in preclinical stages. While simple oxide nanoparticles can be tailored to create ROS bursts during radiotherapy, emerging 2D or porous 3D nanomaterials hold additional potential due to their favorable surface-to-volume ratio facilitating the maximization of catalytic ROS generation. Due to the high freedom in design and the modularity of the synthesis of materials, such as metal organic frameworks, additional functions can be introduced, including drug loading as well as the functionalization with targeting moieties, provided those modifications do not hamper the radioenhancing properties of the materials. Using radioenhancers to reverse a patient's resistance to immune checkpoint inhibitors, is a promising emerging area of research. Immune modulating properties should therefore be understood and leveraged to further maximize cancer treatment efficacy, for example as part of combination therapy settings. However, the efficient translation of nanomaterial-based radioenhancers to the clinical stage is hampered by, among other factors, a lack of standardization of experimental designs and methodologies as well as the absence of direct performance benchmarking. These factors largely preclude data-driven material design. Additionally, the scalable and cost-effective synthesis of high-quality nanomaterials remains challenging for materials other than metal oxides. Comparative studies of different materials using different types of radiation are imperative for a better understanding of the radioenhancing properties of nanomaterials hence enabling the design of performance-optimized radioenhancers for best possible therapy results. This then opens the path to evaluations of the cost-effectiveness of (nanomaterial) radio-enhancement and a careful evaluation of the risk/benefit ratio.

Importantly, the radiation therapy settings, including the type of irradiation and its energy, should also be taken into consideration. At a preclinical stage, low-*Z* NP development can be evaluated with kV X-ray sources, while high-*Z* NPs should always be evaluated with clinical irradiation sources due to differential physical effects. Harnessing advancements from the catalysis community for the nanoparticle design, engineering and analytics in order to achieve performance-optimized generation of X-ray-induced hydroxyl radicals or other ROS species holds promise. While physical and chemical mechanisms are readily accessible *via* simulations or the use of ROS-reactive fluorophores, the understanding of biological radiosensitization mechanisms of NPs in cellular environments requires evaluation in biologically relevant, more complex systems. In addition to the radioenhancement properties, in-depth toxicity evaluations are imperative. Since comparison studies *in vivo* pose ethical concerns, easily accessible preclinical platforms allowing for high-throughput high-content measurements, such as 3D cell models,^276^ should be further integrated into radioenhancer development to gain relevant physical, chemical, and biological data on different nanoparticle candidate materials. Importantly, such advanced *in vitro* models are sufficient for direct performance benchmarking of novel candidate materials against the current clinical gold standard (HfO_2_ nanoparticles), and may hence accelerate clinical translation and reduce animal use.

All in all, recent preclinical and clinical data provide direct evidence for the significant potential of nanomaterials for enhancing clinically established (photon or particle) as well as emerging (*e.g.* FLASH) radiation therapy modalities in an additional way, complementary to developments of advanced instrumentation and treatment planning. This field offers ample opportunities for the material science community to directly contribute to the improvement of radiation therapy by designing performance-optimized radioenhancer nanomaterials.

## Conflicts of interest

There are no conflicts to declare.

## Supplementary Material
